# Characterization of an *in vivo* diode dosimetry system for clinical use

**DOI:** 10.1120/jacmp.v4i2.2528

**Published:** 2003-03-01

**Authors:** Kai Huang, William S. Bice, Oscar Hidalgo‐Salvatierra

**Affiliations:** ^1^ Department of Physics & Astronomy Louisiana State University Baton Rouge Louisiana 70803; ^2^ Division of Radiological Sciences University of Texas Health Science Center San Antonio Texas 78248; ^3^ Mary Bird Perkins Cancer Center 4950 Essen Lane Baton Rouge Louisiana 70809

**Keywords:** *in‐vivo* dosimetry, diode detector, quality assurance

## Abstract

An *in vivo* dosimetry system that uses *p*‐type semiconductor diodes with buildup caps was characterized for clinical use on accelerators ranging in energy from 4 to 18 MV. The dose per pulse dependence was investigated. This was done by altering the source‐surface distance, field size, and wedge for photons. The off‐axis correction and effect of changing repetition rate were also investigated. A model was developed to fit the measured two‐dimensional diode correction factors.

PACS number(s): 87.66.–a, 87.52.–g

## INTRODUCTION

The International Commission on Radiological Units and Measurements (ICRU) recommends that the dose delivered to a tumor be within 5.0% of the prescribed dose.^1^ Each of the many steps in treatment planning and execution will contribute to the overall uncertainty in the dose delivered. The final accuracy of the dose delivered can only be checked directly by means of *in vivo* dosimetry. Because using TLD is labor intensive, and the TLD results cannot be obtained immediately, silicon diode detectors have gained popularity as *in vivo* dosimeters. The main advantage of diodes is that measurements can be obtained on line and allow an immediate check. Other advantages of diodes include high sensitivity, good spatial resolution, small size, simple instrumentation, no bias voltage, ruggedness, and independence from changes in air pressure.^2^

However, just as ion chamber responses are subject to design and environmental aspects, e.g., temperature, atmospheric pressure, etc., silicon diode detector responses are also subject to design and the operating environment. Diodes of different brands must be characterized individually due to different materials and designs. For accurate dosimetry, this characterization needs to be done individually, since even diodes from same batch can be different. Additionally, diodes at different linacs also need to be characterized individually, because the spectra from different linacs might be different even with the same nominal energy.

For any one photon diode detector, the correction factors due to source‐surface distance (SSD) field size, wedge, temperature, beam incident direction, radiation damage, off‐axis distance, etc., need to be characterized. For an electron diode detector, correction factors are dependent upon SSD, cone size, insert, etc.

The aim of the study is to characterize an *in vivo* diode dosimetry system for clinical use during photon irradiation. The method used is entrance dose measurements. Since temperature dependence, directional (angular) response, radiation damage response, etc. of diodes have been extensively studied,^4^ ^–^ ^18^ and the sensitivity of the diode to these effects can usually be obtained from the company's product manuals, this study centered on dose rate dependence and off‐axis corrections. Because the dose per pulse can be altered by SSD, field size, and choice of wedge, they were investigated one by one. A model was developed to fit the measured diode correction factors.

## MATERIALS AND METHODS

The Mary Bird Perkins Cancer Center (MBPCC) has five Linear accelerators. They are Varian 600C, Varian 2100EX (Baton Rouge), Varian 2100C, Varian 2100EX (Covington), Varian 2000CR (Hammond) (Varian Oncology System, Palo Alto, CA). For photons, Varian 600C is used at a single energy: 6 MV, and all other linacs are used at dual energies. The Varian 2100C and Varian 2100EX (Covington) run at 6 and 18 MV; Varian 2100EX (Baton Rouge) at 4 and 10 MV; and Varian 2000CR (Hammond) at 6 and 15 MV. All photon and electron beams are calibrated according to the AAPM TG‐51 protocol, and are calibrated to deliver 1.00 cGy/MU in muscle for a $10\times 10\;{\text{cm}}^{2}$ field size or cone size and 100 cm SSD at the depth of maximum buildup, $d_{\max}$.

The *in vivo* dosimeter (IVD) systems implemented at MBPCC are all IVD Model 1131 (Sun Nuclear Corporation, Melbourne, FL), and all diodes are of *p*‐type, since *p*‐type diodes are generally better than *n*‐type diodes in radiation measurements.^2^ ^,^ ^3^ ^,^ ^19^ Except the Varian 600C, which is equipped with one Sun Nuclear Corporation QED photon diode, each other linac is equipped with two Sun Nuclear Corporation photon diodes. All linacs have Sun Nuclear Corporation QED photon diodes with the exception of the varian 2100C, which has two Sun Nuclear Corporation Isorad‐p photon diodes. Each photon diode is used just for one photon energy and one linac.

The QED photon diodes are constructed with internal build‐up (aluminum or brass) for three energy ranges of 1–4 MV, 6–12 MV, and 15–25 MV, which are color‐coded blue, gold, and red, respectively. All diodes are connected to a dedicated IVD electrometer. The Isorad‐p photon diode detectors are designed with cylindrical symmetry, which can be beneficial in some applications, such as tangential treatments. Besides aluminum and brass, the internal build‐up materials of Isorad‐p still include tungsten. All phantom measurements were made on the RMI $30\times 30\;{\text{cm}}^{2}$ Solid Water (GAMMEX RMI, WI). The diode was taped on the surface of the solid water, with the buildup side facing the beam.

The IVD systems were calibrated by irradiating the diodes under reference conditions (100 cm SSD, $10\times 10\;{\text{cm}}^{2}$ field size or cone size) in the beam. Our diode calibration protocol requires adjustment of the diode reading until it is equal to the dose at the diode with buildup.

The diode correction factor (DCF) used in this study is defined as

(1) DCF=Dose at Diode/Diode Reading.

Since for photons

(2) $$Dose\;Rate=\dot{D}\;\;ref*Sc*Sp*TMR*ISF*WF*OAF,$$

where $\overset{\cdot }{D}\;\text{ref}=1.000\;\text{cGy}/\text{MU}$ at $100+d_{\max}\;\text{cm}$, *Sc* is collimator scatter factor, *Sp* is phantom scatter factor, *TMR* is the tissue maximum ratio, *ISF* is the inverse square factor, *WF* is the wedge factor, and *OAF* is the off axis factor. Generally OAF will not be considered; therefore,

(3) $$DCF=MU*Sc*Sp*{\left[(100+d_{\max})/SSD\right]}^{2}*WF/Diode\;\;Reading.$$

In this study the DCFs are a function of three variables: SSD, FS, and wedge. Typically correction factors are considered linearly independent. For example, if one determines a correction factor for field size at 100 cm SSD, ${\text{DCF}}_{\text{FS}}$, and one for SSD at 10×10 field size (FS) or cone size, ${\text{DCF}}_{\text{SSD}}$, the total diode correction factor for specific FS and SSD is

(4) $$DCF_{SSD\&FS}=DCF_{SSD}*DCF_{FS}.$$

However, the method used in this study does not rely on this assumption. Instead correction factors for FS, ${\text{DCF}}_{\text{FS}}$ were determined for different SSDs instead of just for one SSD. Similarly, ${\text{DCF}}_{\text{SSD}}$ was also found for different FSs instead of for just one FS. So DCF was characterized as an explicit function of SSD, FS and wedge, i.e., ${\text{DCF}}_{\text{FS}\&\text{SSD}}=\text{DCF}$ (SSD, FS) for each wedge. Accordingly two‐dimensional data collection tables were used. An example for open fields (i.e. without wedge) is given in Table I. The same SSDs were used for wedged fields. The same FSs were used up to the maximum FS available for the wedge.

**Table I:** Diode correction factors data collection table for open fields of photons, where 5×5 is the field size in cm^2^, and 70 is the SSD in cm.

	5×5	10×10	20×20	40×40
70
80
90
100
110
120

Based upon experience, it is better to complete one entire group of data (the data in Table I) as quickly as possible. This reduces the error caused by the drift of the diode system. The main source of drift of the diode system is the short life of the system batteries. It was found that the readings of a diode were a function of the available charge of the batteries and that the first several readings of newly recharged system and readings shortly before recharging were not accurate. So it is best to entirely finish one group of data before recharging the batteries.

Unfortunately, the batteries can just last only one to three hours, and recharging was needed several times per day. Therefore, the data from different groups were adjusted to remove the effect of the diode system's drifts. By way of example, suppose we want to adjust the data for open, 15° wedged, 30° wedged, 45° wedged, 60° wedged fields of 2000CR (Ham) 6 MV that were taken over a period of several days. To adjust these data, diode readings are taken in one session with $\text{FS}=10\times 10\;{\text{cm}}^{2},\text{SSD}=100\;\text{cm}$, and MU=300, for open, 15° wedged, 30° wedged, 45° wedged, 60° wedged fields of 2000CR (Ham) 6 MV. Usually these measurements can be finished in less than 10 min, and the drift of the diode system can be neglected. From the five readings obtained above, we can get the ratios between the reading of wedged fields to that of the open field. We assume that the ratios from the data of groups may be inaccurate, and therefore use the ratios from the single session to adjust them.

The off‐axis diode correction was investigated for 4 MV with 60° wedge on the 21EX (BR). The effect of changing the repetition rate of the linacs was also investigated.

## RESULTS AND DISCUSSION

### SSD dependence

**Figure 1:**
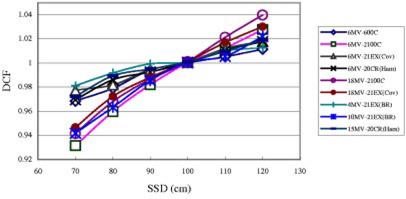
(Color) Diode correction factors as a function of the source to surface distance, SSD, for entrance measurements. All data in this figure are for open fields with field size $10\times 10\;{\text{cm}}^{2}$.

Figure 1 shows that the SSD correction factors for two types of silicon diode detectors, where the 6‐MV–2100C and 18‐MV–2100C are Isorad‐p diode detectors, others are QED *p*‐type diode detectors. All diodes’ DCFs decrease with decreasing SSD. This implies an over response of the diode with increased dose per pulse (decreased SSD). Additionally as the SSD decreases, the number of contamination electrons and head scattered low energy photons able to reach the sensitive part of the diode detector is larger, so the DCF, ratio of ion chamber, reading over diode reading, decreases.^8^ ^,^ ^11^ ^,^ ^12^ For a 10×10 field size, the range for DCF is between 0.93 to 1.04. For small SSD and FS, or large SSD and FS, the range is larger, e.g., DCFs for SSD=70 cm and FS

$=5\times 5\;{\text{cm}}^{2}$, and SSD=120 cm and $\text{FS}=40\times 40\;{\text{cm}}^{2}$, 2100C (BR)'s 18 MV Isorad‐p photon diode, are 0.90 and 1.06, respectively. Generally, the SSD dependence for the Isorad‐p diode is larger than that for QED diode of the same photon energy.^17^ ^,^ ^18^ It was found that for open fields with $10\times 10\;{\text{cm}}^{2}$ field size, all three 6 MV QED diodes’ SSD dependences were within 3% and the differences among them were small (within 1%), but the 6 MV Isorad‐p diode's SSD dependence was up to 7%. However, the SSD dependencies were almost the same for two 18 MV diodes, one was the QED diode, another one was the Isorad‐p diode, for open fields with $10\times 10\;{\text{cm}}^{2}$ field size.

### Field‐size dependence

**Figure 2:**
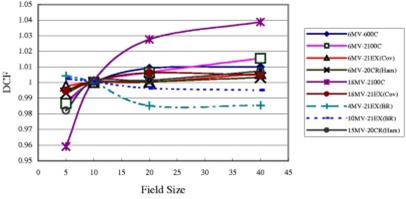
(Color) DCF as a function of the field size for entrance measurements. All data in this figure are for open fields with 100 cm SSD.

Figure 2 shows the DCFs for various field sizes (FSs) for all diodes at SSD 100 cm. Generally the field‐size effect is due to the different irradiation conditions between the diodes and the ion chamber. Since the diode is at the surface, and lacks an overlaying layer, its reading is less dependent upon the phantom scatter, and heavily dependent on head scatter. Therefore, DCF increases as the diode under responds with increase in field size.^14^ ^,^ ^8^ ^,^ ^11^ ^,^ ^12^ This happened for the majority of diodes used at MBPCC, except the two QED diodes for the 21EX (BR), one for 4 MV and another for 10 MV. In fact, the 4 MV diode showed the opposite behavior, i.e., DCF decreased and diode over responded with increase of field size. For the 10 MV diode, the DCF roughly remained a constant when FS changed. Other authors have noted a similar phenomenon for the Scanditronix 18 MV EDP30 diode, explaining that the build‐up cap was not thick enough to guarantee electronic equilibrium.^11^ Some electrons scattered from the accelerator head may have reached the sensitive part of the diodes. In order to check this assumption, we used a small piece of solid water (5 mm thick) on the top of the 4 MV QED diode, and then re‐made the measurements. Because this time the total buildup was the solid water plus the inherent buildup of the diode, the total buildup thickness was greater than $d_{\max}$. However, the FS dependence of this 4 MV QED diode was still as described previously, i.e., the DCF decreased with increasing FS. This casts doubt on the assumption of insufficient buildup. Two gold QED diodes and one gold Isorad‐p

photon diode, all designed for 6–12 MV photons, were also used to replace the existing 4 MV QED diode to re‐measure the 4 MV photon beams of linac 21EX (BR). It was found that the Isorad‐p diode behaved normally, i.e., the DCF increased with increase of FS. But all gold QED diodes showed the opposite behavior, similar to the blue (1–4 MV) diode. Similarly, these two gold (6–12 MV) QED diodes and one Isorad‐p diode were used to measure 10 MV photon beams of linac 21EX (BR). It was found that the Isorad‐p diode behaved normally, both gold QED diodes showed the opposite behavior. However, these gold QED diodes behave normally when they are used for 6 MV photons from other linacs. Two small pieces of solid water (5 mm thick each) were also put under the 4 and 10 MV QED diodes to mimic the geometry of Isorad‐p type diodes, but the results were still abnormal. It seems that both linac and diode itself contributed to the abnormal behavior at 4 and 10 MV.

It was also noted that the 18 MV Isorad‐p photon diode on the 2100C is much more dependent on field size than other diodes. The change is up to 8% when field size changes from $5\times 5\;{\text{cm}}^{2}$ to $40\times 40\;{\text{cm}}^{2}$ for open fields. But the FS dependence of 6 MV Isorad‐p photon diode at 2100C is smaller and is similar to those of QED diodes.

### Wedge effects

**Figure 3:**
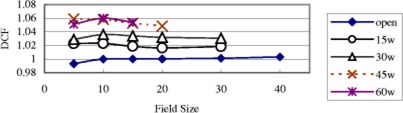
(Color) DCF of a 6 MV QED diode on the linac 20CR (Ham) as a function of the field size for entrance measurements. All data in this figure are for SSD 100 cm.

**Figure 4:**
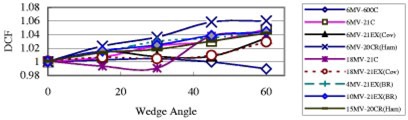
(Color) DCF as a function of the wedge angle for entrance measurements. All data in this figure are for SSD 100 cm and FS $10\times 10\;{\text{cm}}^{2}$, and only for narrow and upper wedges.

Figure 3 shows the field‐size dependence of the 6 MV QED photon diode of 2000CR (Ham). The DCF of this diode doesn't change significantly when the field size changes. From this figure, one can see that DCF increases with wedge angle. This is because dose per pulse decreases with increasing wedge angle, and from Fig. 1, the decrease in dose per pulse (i.e., increase in SSD) leads to an increase in DCF. Although generally the case, it was found that DCF does not always increase with increasing wedge angle;^8^ see Fig. 4.

**Figure 5:**
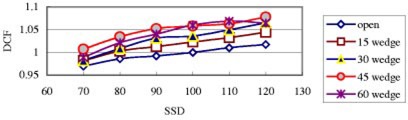
(Color) Diode correction factors as a function of the SSD for entrance measurements [$10\times 10\;{\text{cm}}^{2}$ field size and a 6 MV QED photon diode on the linac 20CR (Ham)].

**Figure 6:**
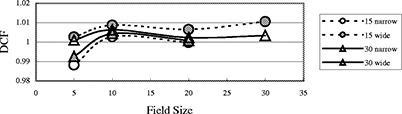
DCF of a 6 MV QED diode on the linac 600C (BR) as a function of the field size for entrance measurements (100 cm SSD, 15°/30° narrow and wide wedges).

**Figure 7:**
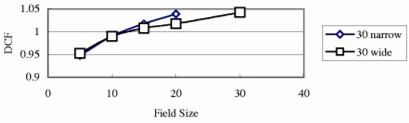
(Color) DCF of an 18 MV Isorad‐p diode on the linac 2000C (BR) as a function of the field size for entrance measurements (100 cm SSD, 30° narrow and wide wedges).

Figure 5 shows the SSD dependence of open and wedged fields for a 6 MV QED diode. It can be seen that wedges do not change the general shape of the curves. Generally the DCF difference between narrow and wide wedges for the same degree is small (within 1%, Fig. 6). For the 18 MV Isorad‐p diode, the DCF difference is up to 2% (Fig. 7). However, the DCF difference between the upper wedged beam and the lower wedged beam is somewhat large, especially for small SSDs.

**Figure 8:**
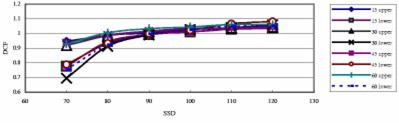
(Color) The SSD dependence for upper and lower wedged beams with $10\times 10\;{\text{cm}}^{2}$ field size. The diode is a 4 MV QED photon diode on the linac 21EX (BR).

Figure 8 shows the DCFs for upper and lower wedged fields. There are two groups of DCF curves: the upper one is for upper wedged fields, and the lower one is for lower wedged fields. The difference between two groups is generally 10% and up to 20% for 70 cm SSD. This is due to different distances between wedges and the diode for upper and lower wedges.

**Figure 9:**
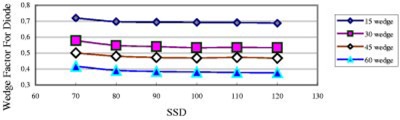
(Color) Wedge factors for diodes as a function of the SSD for entrance measurements [field size $10\times 10\;{\text{cm}}^{2}$, QED 6 MV photon diode on the linac 21EX (COV)].

Figure 9 shows the “wedge factors” for diode. They are not the ordinary wedge factors measured using the ion chamber. The wedge factor for diodes used here is the ratio of diode reading with wedge over that without wedge. It is apparent that wedge factors for diode decrease with increase of SSD.

### Two‐dimensional DCF

**Figure 10:**
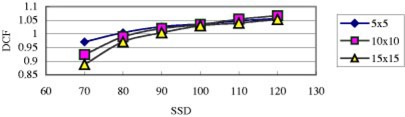
(Color) SSD dependence of a QED 6 MV photon diode on the linac 21EX (COV) for different FSs (all for 60° wedged fields).

**Figure 11:**
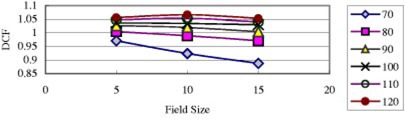
(Color) FS dependence of a QED 6 MV photon diode on the linac 21EX (COV) for different SSDs (all for 60° wedged fields).

As previously described, an uncorrected diode response may overestimate or underestimate the dose, dependent on FS and SSD. That is, the SSD dependence of DCF $\left({\text{DCF}}_{\text{SSD}}\right)$ is a function of FS (Fig. 10), and similarly FS dependence of DCF $\left({\text{DCF}}_{\text{FS}}\right)$ is a function of SSD (Fig. 11). Thus, ${\text{DCF}}_{\text{FS}}*{\text{DCF}}_{\text{SSD}}$ is not necessarily equal to ${\text{DCF}}_{\text{FS}\&\text{SSD}}$, especially for wedged fields. Therefore, we used a two‐dimensional method to measure and model ${\text{DCF}}_{\text{FS}\&\text{SSD}}$. For illustration consider first the situation of FS=5×5 and SSD=70. From Figs. 10 and 11, one gets for the 60° wedged fields, ${\text{DCF}}_{\text{FS}=5\times 5}=1.040$ for SSD=100, and ${\text{DCF}}_{\text{SSD}=70}=0.924$ for FS=10×10. Then, ${\text{DCF}}_{\text{FS}}*\;{\text{DCF}}_{\text{SSD}=}0.961$. However, from the figures above, ${\text{DCF}}_{\text{FS}=5\times 5\&\text{SSD}=70}=0.971$. The difference between ${\text{DCF}}_{\text{FS}}*\;{\text{DCF}}_{\text{SSD}}$ and ${\text{DCF}}_{\text{FS}\&\text{SSD}}$ is insignificant (1%). In contrast, consider another situation with FS=15×15 and SSD=70. ${\text{DCF}}_{\text{FS}=15\times 15}=1.030$ for SSD=100, and ${\text{DCF}}_{\text{SSD}=70}=0.924$ for FS=10×10. Then ${\text{DCF}}_{\text{FS}}*\;{\text{DCF}}_{\text{SSD}}=0.952$. The value for ${\text{DCF}}_{\text{FS}=15\times 15\&\text{SSD}=70}$ is 0.888. The difference is now about 7%.

### Off‐axis correction

In order to find the range of the off‐axis effects, off‐axis diode corrections were investigated for 4 MV with a 60° wedge at linac 21EX (BR). The off‐axis correction is defined as

(5) Off−axis correction=(OAF of diode)/(OAF of ion chamber),

where OAF means off‐axis factor. The off‐axis correction measured in the wedge direction was within 1.5%. Thus, the off‐axis correction of diode can usually be neglected and one may use the OAF from ion chamber measurements tabulated in the dosimetry book directly. However, since a displacement of the diode in the direction of the wedge profile of 1.0 cm results in a 9% error for this 4 MV with 60° wedge, and since it is difficult to put the diode detector at the central axis accurately, a larger tolerance should be considered for wedged fields when performing *in vivo* dosimetry.

The off‐axis correction above is for displacement of the diode in the direction of the wedge profile. The off‐axis correction for displacement of the diode in the direction perpendicular to the direction of the wedge profile was also measured, for the 4 MV diode and 60° wedged field on the 21EX (BR), with 100 cm SSD and $15\times 15\;{\text{cm}}^{2}$ FS. It was found that the off‐axis correction in this direction could be neglected (within 0.5%).

### Effect of changing repetition rate

Sometimes the linac's repetition rates are changed from default values. We also investigated the relative response between the ion chamber (i.e., output of linac) and the diode. We chose 4 and 10 MV of the 21EX (BR) and 6 MV of the 600C (BR). It was found that there was no difference between responses of ion chambers and diodes, i.e., for changes in repetition rate (in MU/min), no correction was needed to correct the diode's reading to the ion chamber's reading. For 6 MV of the 600C (BR) and 10 MV of the 21EX (BR), both the ion chamber's reading and the diode's reading remained unchanged when the repetition rate changed. However, for 4 MV of the 21EX (BR), both the ion chamber's reading and the diode's reading changed, at the same ratio, when the repetition rate changed. For example, when repetition rate changed from 250 MU/min to 50 MU/min, both the ion chamber's reading and the diode's reading increased 2%.

### Data fitting

Our general approach was to find physically meaningful parameters, then perform a least squares fitting to describe the DCF in terms of these parameters. To fit the data for a specific diode on a specific linac with a specific energy, two corrections were introduced: field size correction and wedge correction. A second order polynomial was used to model the field size corrections. That is,

(6) $$FS\;correction=b2*FS^{2}+b1^{*}FS+b0,$$

where FS is the equivalent field size at 100 cm SSD in cm.

It's desirable to put all data, open and wedged fields, together into a single model. Then the wedge corrections need to be introduced. Since the wedge factor (not the wedge correction here) decreases with increase in SSD (Fig. 9), a constant wedge correction is not enough to describe the real diode wedge factor shown in Fig. 9. Thus the following second order polynomial was used to model the wedge corrections:

(7) $$Wedge\;correction=WF*[w2*{\left(100/SSD\right)}^{2}+w1*(100/SSD)+w0],$$

where the *WF* takes different value for different wedge angles. *WF* is named as *WF*15, *WF*30, *WF*45, *WF*60 for 15°, 30°, 45°, 60° wedges, respectively. The narrow wedge and wide wedge of the same degree have the same *WF* value, e.g., *WF*15 is for both narrow and wide 15° wedges, since the difference between them is small (within 2%). This method worked well for all linacs except 21EX (BR) [only upper wedges are in use for another 21EX linac, 21EX (Cov)], since the DCF difference between upper wedged beam and lower wedged beam is somewhat large, especially for small SSDs (Fig. 8). So finally eight *WFs* were introduced to fit the data: *WF*15*u*, *WF*30*u*, *WF*45*u*, *WF*60*u*, *WF*15*l*, *WF*30*l*, *WF*45*l*, *WF*60*l*, where “*u*“ represent upper and ‘*l*’ represent lower. (Alternatively, we can fit data of upper and lower wedges separately.) The *w*2, *w* 1, and *w*0 are the same for all wedged beams for a specific photon diode and a specific photon energy. Using 100/SSD instead of SSD in above wedge correction formula describes the decrease of the diode wedge factor with increase of SSD (Fig. 9).

A parameter named lambda was introduced. Lambda is essentially dose per pulse at the diode with the field correction and wedge correction included. That is,

(8) $$Lambda={\left[(100+d_{\max})/SSD\right]}^{2}*(FS\;correction)*(Wedge\;correction).$$

Finally a fit was performed using the method of least square error and the curve relating DCF and lambda was found. The fitting polynomial is of the form

(9) $$DCF=a0+a1*Lambda+a2*{\left(Lambda\right)}^{2}+a3*{\left(Lambda\right)}^{3}.$$

**Figure 12:**
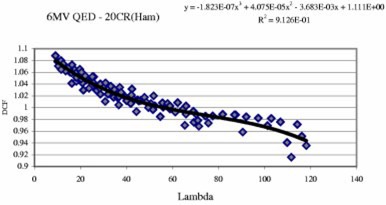
(Color) The fitted curve and polynomial of a 6 MV QED diode on the linac 2000CR (Ham).

Generally a second order polynomial is sufficient, since the difference between second and third order polynomial fits is usually less than 1% over the clinical useful range. The fitting was done using Microsoft excel by adjusting all fourteen or eighteen variables listed above (i.e. *b*2, *b*1, *b*0, *WF*15, *WF*30, *WF*45, *WF*60, *w*2, *w*1, *w*0, *a*0, *a*1, *a*2, and *a*3). This is an optimization problem with 14 or 18 variables listed above and many optimization routines can be used to resolve it. The fitting results are shown in Table II. Figure 12 shows an example of the fitted curve and polynomial. $R^{2}$ is the coefficient of determination. A similar method can also be applied to electron diodes.

**Table II:** Fitting results.

Energy, Diode & Linac	6MV QED 600C (BR)	4MV QED 2100EX (BR)	10MV QED 2100EX (BR)	6MV Isorad‐p 2100C (BR)	18MV Isorad‐p 2100C (BR)	6MV QED 2100EX (COV)	18MV QED 100EX (COV)	6MV QED 2000CR (Ham)	15MV QED 2000CR (Ham)
*b*2	−1.087E−3	−0.01458	−0.00573	−3363E−4	0.004164	−0.007071	−0.001235	−0.02650	3.060E−4
*b*1	0.05231	0.76361	0.27829	0.008931	−0.26328	0.40266	0.05640	1.41491	−0.01553
*b*0	1.19027	7.53744	7.58613	1.69969	7.22563	1.72589	1.7919	36.65421	1.07815
*WF*15	0.003461			0.007402	0.01528	0.73015	0.55297	0.002650	0.003257
*WF*30	0.003924			0.006946	0.01546	1.09422	0.62387	0.002504	0.003371
*WF*45	0.004108			0.006910	0.01138	0.94316	0.57349	0.001491	0.002679
*WF*60	0.004761			0.006024	0.01012	0.77166	0.48187	0.001695	0.002451
*WF*15*u*		0.003292	0.002930						
*WF*151		0.005116	0.004295						
*WF*30*u*		0.003650	0.003135						
*WF*30*l*		0.006112	0.004836						
*WF*45*u*		0.003058	0.002646						
*WF*45*l*		0.004859	0.004742						
*WF*60*u*		0.002972	0.002602						
*WF*60*l*		0.005981	0.005173						
*w*2	103.45	221.07	221.06	−2.6892	−24.882	−1.4418	−1.6466	103.07	103.07
*w*1	75.757	37.197	37.171	38.764	27.7205	6.5061	6.6566	75.868	75.868
*w*0	64.637	−83.01	−83.049	81.2555	78.817	−4.1401	−3.4922	65.138	65.138
*a*0	1.0375	1.058	1.068	1.2158	1.1555	1.0386	1.0576	1.1106	1.0714
*a*1	−0.02861	−2.081E−3	−6.558E−3	−0.1966	−0.03191	−6.018E−3	−0.02270	−3.683E−3	−9.300E−2
*a*2	0.003858	−6.452E−5	−6.227E−6	0.05427	9.243E−4	5.494E−5	3.688E−5	4.075E−5	2.795E−2
*a*3	−3.571E−4	5.186E−7	0.000	−6.046E−3	0.000	−1.128E−06	0.000	−1.823E−7	−3.948E−3
*R*‐	0.8769	0.9170	0.9481	0.9417	0.9413	0.9301	0.9409	0.9126	0.8584
squared value (*R* ^2^)									

## CONCLUSION

In this study, an *in vivo* dosimetry system that uses *p*‐type semiconductor diodes with buildup caps was characterized for clinical use. The dose per pulse dependence was investigated. This was done by altering the SSD, field size and wedge for photons. Because ${\text{DCF}}_{\text{FS}}*\;{\text{DCF}}_{\text{SSD}}$ is not necessarily equal to ${\text{DCF}}_{\text{FS}\&\text{SSD}}$, two‐dimensional data collection and modeling were used.

For SSD dependence of open fields with 10×10 field size, the range for DCF is between 0.93 to 1.04, i.e., within 7%. For small SSD and FS, or large SSD and FS, the range is larger, e.g., DCFs for SSD=70 cm and $\text{FS}=5\times 5\;{\text{cm}}^{2}$, and SSD=120 cm and $\text{FS}=40\times 40\;{\text{cm}}^{2}$, 2100C (BR)'s 18 MV Isorad‐p photon diode, are 0.90 and 1.06, respectively.

For FS dependence of open fields with 100 cm SSD, the range for DCF is generally within 2%, i.e., from 0.98 to 1.02. But for the 18 MV Isorad‐p diode on the 21EX (BR), the range is larger (0.96 to 1.04). Generally, the SSD dependence for Isorad‐p diode is larger than that for QED diode of the same photon energy.

The DCF for a wedged field is generally larger than that for corresponding open field, since dose per pulse becomes lower for a wedged field. The DCF difference between narrow and wide wedges with same degree is small, generally within 1% but up to 2% for 18 MV Isorad‐p diode. However, the DCF difference between upper wedged beam and lower wedged beam is large, especially for small SSDs. The difference is generally 10% and up to 20% for 70 cm SSD. This is due to different distances between wedges and the diode for upper and lower wedges.

The off‐axis correction and effect of changing repetition rate of linac were also investigated. It was found that the off‐axis correction was within 1.5%. Thus, the off‐axis correction of diode can usually be neglected and one may use the OAF from ion chamber measurements tabulated in the dosimetry book directly. It was found that there was no difference between responses of ion chambers and diodes when repetition rate (in MU/min) was changed, i.e., no correction is needed to correct the diode's reading to the ion chamber's reading.

A model was created to fit the measured diode correction factors. The basic idea was to find physically meaningful parameters, and then perform a least squares fitting to describe the data. The fitting results can be used clinically.
